# Examining the Role of Low Temperature in Satsuma Mandarin Fruit Peel Degreening *via* Comparative Physiological and Transcriptomic Analysis

**DOI:** 10.3389/fpls.2022.918226

**Published:** 2022-07-13

**Authors:** Oscar W. Mitalo, William O. Asiche, Seung W. Kang, Hiroshi Ezura, Takashi Akagi, Yasutaka Kubo, Koichiro Ushijima

**Affiliations:** ^1^Graduate School of Life and Environmental Sciences, University of Tsukuba, Tsukuba, Japan; ^2^Graduate School of Environmental and Life Science, Okayama University, Okayama, Japan; ^3^Department of Research and Development, Del Monte Kenya Ltd, Thika, Kenya; ^4^Tsukuba Plant Innovation Research Center, University of Tsukuba, Tsukuba, Japan

**Keywords:** chlorophyll, citrus, degreening, ethylene, RNA-Seq, on-tree, storage

## Abstract

Peel degreening is the most conspicuous aspect of fruit ripening in many citrus fruits because of its importance for marketability. In this study, peel degreening in response to propylene (an ethylene analog) and at varying storage temperatures was characterized in Satsuma mandarin (*Citrus unshiu* Marc.) fruit. Propylene treatment triggered rapid peel degreening (within 4–6 days), indicated by an increase in the citrus color index (CCI) and chlorophyll loss. Peel degreening was also observed in fruit at 10°C and 15°C after 28–42 days, with gradual CCI increase and chlorophyll reduction. However, fruit at 5°C, 20°C, and 25°C remained green, and no substantial changes in peel CCI and chlorophyll content were recorded during the 42-day storage duration. The transcriptomes of peels of fruit treated with propylene for 4 days and those stored at varying temperatures for 28 days were then analyzed by RNA-Seq. We identified three categories of differentially expressed genes that were regulated by (i) propylene (and by analogy, ethylene) alone, (ii) low temperature (5°C, 10°C, or 15°C vs. 25°C) alone, and (iii) either propylene or low temperature. Gene-encoding proteins associated with chlorophyll degradation (such as *CuSGR1, CuNOL, CuACD2, CuCAB2*, and *CuLHCB2*) and a transcription factor (*CuERF114*) were differentially expressed by propylene or low temperature. To further examine temperature-induced pathways, we also monitored gene expression during on-tree fruit maturation vs. postharvest. The onset of on-tree peel degreening coincided with autumnal drops in field temperatures, and it was accompanied by differential expression of low temperature-regulated genes. On the contrary, genes that were exclusively regulated by propylene (such as *CuCOPT1* and *CuPOX-A2*) displayed insignificant expression changes during on-tree peel degreening. These findings indicate that low temperatures could be involved in the fruit ripening-related peel degreening independently of ethylene.

## Introduction

During ripening, citrus fruits undergo a conspicuous color change in the peel from green to yellow, red, or orange (Iglesias et al., 2007). This color change, commonly referred to as peel degreening, is mainly caused by the successive degradation of two major green-colored chlorophyll (Chl) pigments, namely, Chl a and Chl b, to colorless compounds (Hörtensteiner, 2006). To enter into the degradation pathway, Chl b is first converted to Chl a by Chl b reductase, which is encoded by *non-yellow coloring 1* (*NYC1*) and *NYC1-like* (*NOL*) (Horie et al., 2009). Key steps in the Chl a degradation pathway include the dephytilation by chlorophyllase (CLH) and pheophytinase (PPH) and the removal of the central Mg atom by Mg dechelatase, which is encoded by *stay-green* (*SGR*) (Shimoda et al., 2016). The resulting compound pheophorbide is then converted to red Chl catabolites (RCC) by pheophorbide a oxidase, and RCC is reduced to colorless compounds by RCC reductase (RCCR), which is encoded by *accelerated cell death 2* (*ACD2*) (Mach et al., 2001). In addition, Chl degradation is also facilitated by the disaggregation of photosystem proteins, particularly the light-harvesting complex (LHC) and Chl a–b binding (CAB) proteins (Barry, 2009), which leads to the accumulation of free Chls and hence allows for easier access by degrading enzymes. Several transcription factors, including ethylene response factors (ERFs), directly influence Chl degradation-related genes (Yin et al., 2016), and their expression levels have been shown to negatively correlate with Chl content in citrus fruit peels (Xie et al., 2014).

It has been broadly demonstrated that exogenous ethylene application accelerates peel degreening in citrus fruits (Mayuoni et al., 2011), despite being classified as non-climacteric. In this regard, ethylene treatments are routinely used in the citrus fruit industry to hasten the degreening process or to ensure uniform color (Porat, 2008; Morales et al., 2020). Exogenous ethylene activates the expression of various genes encoding Chl degradation enzymes (Shemer et al., 2008; Yin et al., 2016), as well as transcription factors (Yin et al., 2016). However, the role of endogenous ethylene in natural peel degreening is still puzzling since mature citrus fruits produce trace ethylene levels (Katz et al., 2004).

Low temperature has also been shown to promote peel degreening in several citrus fruit species including lemons (Manera et al., 2012; Conesa et al., 2019; Mitalo et al., 2020), grapefruits (Manera et al., 2013), oranges (Carmona et al., 2012a), and mandarins (Tietel et al., 2012). However, there is a conflicting information regarding the regulatory mechanisms involved. On one hand, it is argued that the low ethylene amounts produced by mature citrus fruits are likely involved in low temperature-induced peel degreening (Goldschmidt et al., 1993; Carmona et al., 2012b). However, our recent study in lemons (Mitalo et al., 2020) showed that low-temperature enhancement of peel degreening is achieved by the activation of genes associated with Chl degradation, carotenoid metabolism, photosystem proteins, and transcription factors, independently of endogenous ethylene. Ethylene-independent stimulation of fruit ripening by low temperatures has also been reported in other fruit species such as kiwifruit (Asiche et al., 2018; Mitalo et al., 2019) and apples (Tacken et al., 2010). In citrus fruit, however, it remains unclear whether a similar phenomenon takes place in other species besides lemons or not, as some species/varieties are known to be more sensitive to ethylene than others (Petracek and Montalvo, 1997; John-Karuppiah and Burns, 2010; Alós et al., 2014).

In Japan, citrus cultivation is predominated by Satsuma mandarin (*Citrus unshiu* Marc.) fruit (Omura and Shimada, 2016), which comprises numerous cultivars that account for 62.5% of all citrus acreage^1^ During fruit maturation, especially in the late-harvesting genotypes, peel degreening often begins in October (Takagi et al., 1994; Ikoma et al., 2001), in conjunction with the ambient low temperatures that are associated with autumn. Additionally, most early-maturing genotypes, which are usually harvested in August (during high summer temperatures), typically display poor peel coloration (personal observation). These findings suggest that low temperatures are required for normal color development in Satsuma mandarins, but the phenomenon is yet to be investigated objectively.

In this study, we examined whether low temperature had a direct role in peel degreening during natural maturation in Satsuma mandarin fruit. Postharvest peel color changes were monitored in response to propylene (a well-known ethylene analog; McMurchie et al., 1972) treatment at 25°C or during storage at 5°C, 10°C, 15°C, 20°C, and 25°C, as well as during on-tree maturation. RNA-Seq was then used to find genes that were differentially regulated by ethylene signaling and/or low temperature. Furthermore, gene expression changes during postharvest and on-tree peel degreening were compared to determine the roles of low temperature and ethylene in their regulation. Overall, data in this study demonstrate that low temperature is deeply involved in the regulation of peel degreening in Satsuma mandarins, possibly *via* an ethylene-independent mechanism.

## Materials and Methods

### Plant Material and Treatments

Satsuma mandarin (*Citrus unshiu* Marc. cv. “Aoshima”) fruit grown under standard cultural practices was harvested in 2014, 2015, and 2017 from a commercial orchard in Takamatsu, Japan. Sorting was carried out to ensure uniform size and color and to remove damaged fruit. Fruit harvested at 145 days after full bloom (DAFB) with green peel and undetectable ethylene levels were used to assess the postharvest effects of ethylene and storage temperature. Two groups of 40 fruits each were used to characterize ethylene-induced peel degreening. The first group of fruits was put in a sealed container and continuously treated with propylene (5,000 μl L^−1^) to induce ethylene signaling and to allow for accurate determination of endogenous ethylene produced by the fruit. The other group of fruits (control) was held in identical containers without propylene treatment. Both treatments (ethylene and control) were carried out for up to 6 days at 25°C. Soda lime was placed in the sealed containers to reduce CO_2_ accumulation. To determine the effect of postharvest storage temperature, five groups of 40 fruits each were stored at either 5°C, 10°C, 15°C, 20°C, or 25°C for up to 42 days. Routine ethylene measurements (twice a week) were carried out during the storage duration. On-tree peel degreening changes were assessed from seven successive harvests in 2017: September 6, September 20, October 5, October 21, November 7, November 18, and December 6, which corresponded to 130, 144, 159, 175, 193, 204, and 222 DAFB. Ethylene measurements were carried out before color determination and sampling. In all experimental setups, fruit peel (flavedo) samples of three replicate fruits were collected, frozen in liquid nitrogen, and stored at −80°C for future analysis.

### Ethylene Measurements

Individual fruits were incubated for 1 h, at their respective storage temperatures, in a 440-ml airtight container. Ethylene production was then determined by injecting 1 ml of headspace gas into a gas chromatograph (Model GC4 CMPF, Shimadzu, Kyoto, Japan), which was equipped with a flame ionization detector (set at 200°C) and an activated alumina column (set at 80°C; Mitalo et al., 2019). This procedure has a minimum ethylene detection capacity of 0.01 nl g^−1^ h^−1^.

### Determination of Peel Color and Chl Content

Peel color measurements were carried out on four evenly distributed equatorial sites using a CR-200B chromameter (Konica Minolta, Tokyo, Japan). Citrus color index (CCI) was calculated from the *L, a*, and *b* Hunter lab parameters (Jiménez-Cuesta et al., 1981), using the *1000a/Lb* transformation (Ríos et al., 2010), and then expressed as the mean value of six replicate fruit. Extraction and quantification of Chls were based on the procedure described in Rodrigo et al. (2003), with slight modifications. Briefly, Chls were extracted in 80% acetone from the flavedo tissue of six replicate fruits collected in 2017, and appropriate dilutions were used to quantify absorbance at 646.8 nm and 663.2 nm. These measurements were then used to calculate Chl content using the Lichtenthaler and Wellburn equations (Wellburn, 1994).

### Library Construction and RNA Sequencing

Samples for RNA-Seq analysis were collected in 2015 immediately after harvest (145 DAFB, 0 days) and 4 days of propylene treatment, as well as after 28 days of storage at 5°C, 10°C, 15°C, 20°C, and 25°C. Total RNA was extracted from ~2 g ground flavedo tissue of three replicate fruit using the phenol-chloroform method (Ikoma et al., 1996), with slight modifications. Treatment with DNase I (Nippon Gene, Tokyo, Japan) was carried out to remove genomic DNA contamination before further purification with FavorPrep after Tri-Reagent RNA Clean-up Kit (Favorgen Biotech. Co., Ping-Tung, Taiwan). Illumina paired-end libraries were then constructed using a NEBNext^®^ Ultra^™^ RNA Library Prep Kit for Illumina (New England Biolabs), and sequencing was performed on an Illumina HiSeq 2500 platform (Hokkaido System Co. Ltd., Japan). Sequenced reads were trimmed to obtain ≥10 million paired reads per sample and to exclude low-quality sequences (Phred score < 20) as well as adapter sequences (Mitalo et al., 2020).

### Differential Gene Expression Analysis

The trimmed reads were mapped to the reference *Citrus clementina* Genome v1.0 (Wu et al., 2014), and mapped reads were counted using CLC genomic workbench (Qiagen, Aarhus, Denmark) with default settings. Gene expression levels were then normalized as reads per kilobase million (RPKM). To determine the effect of ethylene, propylene-treated samples were compared with at-harvest, while the low-temperature effect was assessed using samples obtained at 25°C as a control. Three criteria were used to detect differentially expressed genes (DEGs): (i) RPKM ≥ 3.0 in either of the three replicates, (ii) false discovery rate ≤ 0.01, and (iii) 3-fold increase or decrease in expression levels. Clusters of highly correlated genes were generated using the weighted gene co-expression network analysis (WGCNA) method (Langfelder and Horvath, 2008), with 12 and 0.15 selected as thresholding power and tree-cut parameters, respectively. Significantly enriched gene ontology (GO) terms were established using the agriGO (v.2.0) web-based toolkit (Tian et al., 2017), and hypergeometric tests followed by a Bonferroni correction were used to calculate *P*-values for each term. The cutoff for a significantly enriched GO term was *P* < 0.05.

### Quantitative Real-Time PCR (RT-qPCR) Analysis

Quantitative real-time PCR analysis was carried out on flavedo samples collected in 2017 according to our previous method (Mitalo et al., 2020). Briefly, 2.4 μg of clean RNA was reverse-transcribed to first-strand cDNA using a Takara RNA PCR™ kit (Takara, Shiga, Japan). The gene-specific primers (Supplementary Table 1) were designed using the Primer3 online software (version 0.4.0^2^ The gene expression of three biological replicates was examined on a MYiQ Single-Color Reverse Transcriptase-Quantitative PCR Detection System (Bio-Rad, Hercules, CA, USA) using TB Green^™^ Premix ExTaq^™^ II (Takara, Shiga, Japan). *CuActin* (*Ciclev10025866m*.g) was used as the housekeeping gene after examining its constitutive expression pattern from the RNA-Seq data. Relative expression values were calculated using the 2^−ΔΔCt^ method with at-harvest (144 DAFB, 0 days) samples calibrated as 1.

### Statistical Analysis

Data obtained in this study were subjected to statistical analysis using R v.3.4.0^3^. Differences in CCI, Chl content, and gene expression levels were determined using ANOVA followed by Tukey's *post-hoc* tests.

## Results

### Effect of Propylene Treatment on Peel Degreening

**Figure 1:**
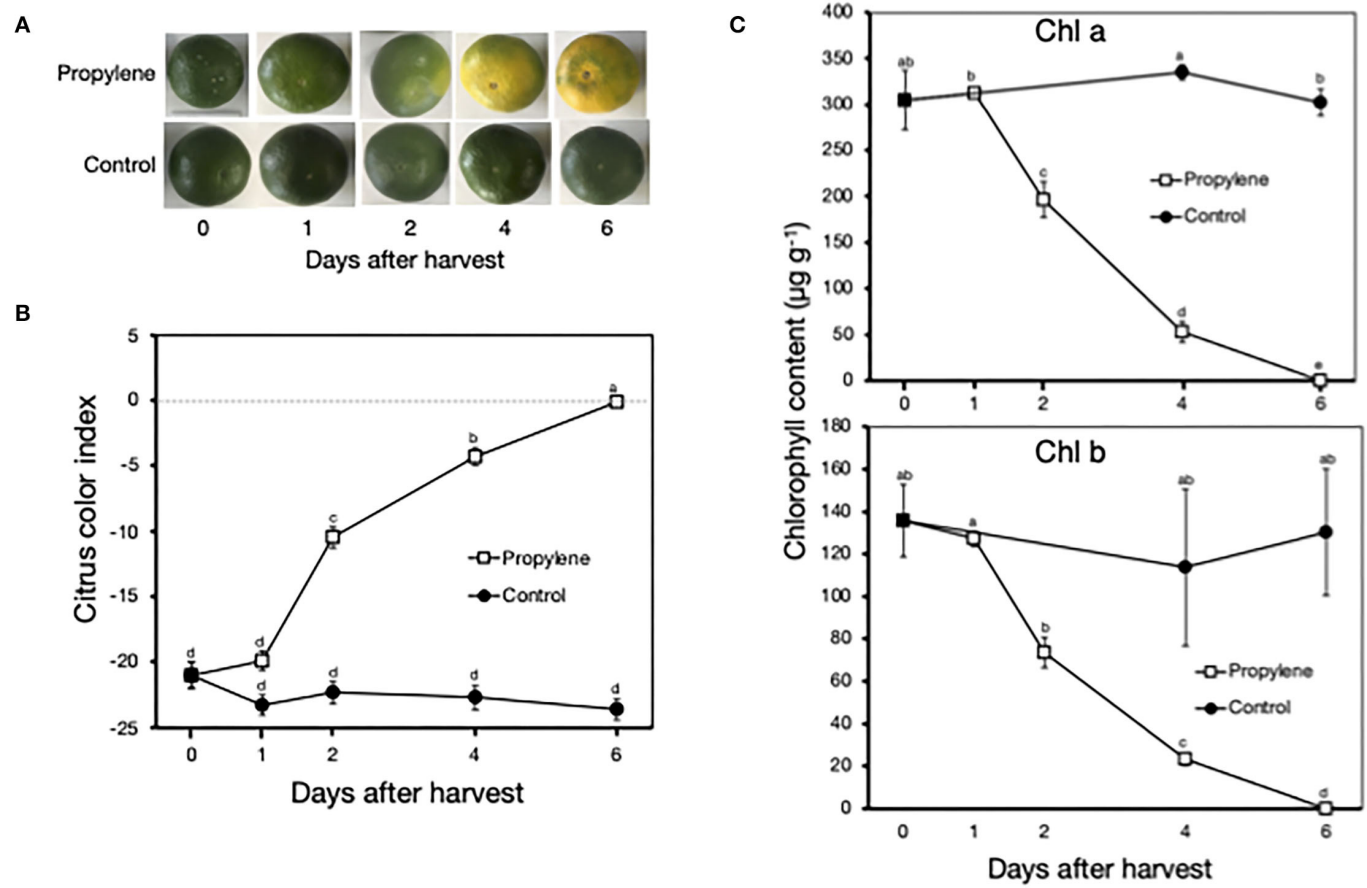
Characterization of ethylene-induced peel degreening in postharvest Satsuma mandarin fruit. **(A)** Appearance of fruit during continuous treatment with propylene, an analog of ethylene. **(B)** Changes in citrus color index. Dashed line indicates color index 0, which represents a color change from green to yellow/orange. **(C)** Changes in peel chlorophyll content. Propylene treatment was carried out continuously at 25°C to induce ethylene signaling. Each data point represents the mean (±SE) of six replicate fruit. Different letters indicate significant differences in ANOVA (Tukey's test, *P* < 0.05).

Propylene treatment triggered a color change in the peel of Satsuma mandarin fruit from green to yellow after 4 and 6 days (Figure 1A), which was indicated by a rapid increase in CCI from −21.0 at harvest to −4.3 at 4 days and −0.1 at 6 days (Figure 1B). In accordance with the loss of green color in propylene-treated fruit, Chl a and Chl b content decreased from the initial at-harvest values of 300 μg g^−1^ and 135 μg g^−1^, respectively, to almost undetectable levels at 6 days (Figure 1C). Non-treated control fruit remained green, showed insignificant changes in CCI, and maintained high Chl a and Chl b content throughout the experimental period.

### Effect of Storage Temperature on Peel Degreening

**Figure 2:**
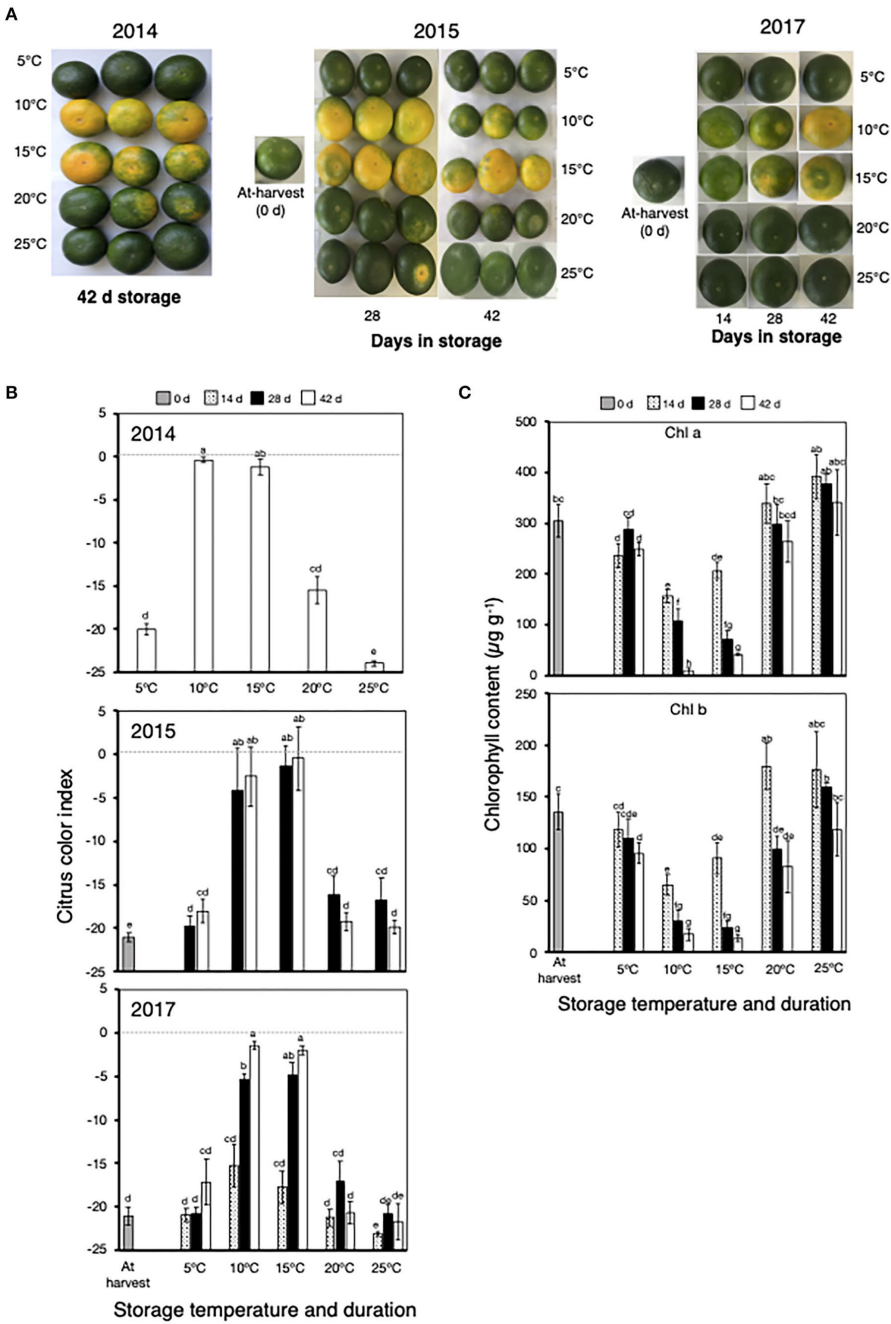
Peel degreening behavior of Satsuma mandarin fruit at different storage temperatures. **(A)** Appearance of fruit during and after storage at different temperatures. **(B)** Changes in citrus color index during and after storage at different temperatures. Dashed line indicates color index 0, which represents a color change from green to yellow/orange. **(C)** Changes in peel chlorophyll content during storage at different storage temperatures. Samples for chlorophyll content determination were collected in 2017. Each data point represents the mean (±SE) of six replicate fruit. Different letters indicate significant differences in ANOVA (Tukey's test, *P* < 0.05).

In 2014, 2015, and 2017, fruit stored at 10°C and 15°C showed an appreciable change in peel color from green to yellow after 28 and 42 days (Figure 2A), with a concomitant increase in CCI from the initial at-harvest values of about −21.0 to approximately −5.0 at 28 days and near-zero values at 42 days (Figure 2B). There was also a substantial decrease in the content of both Chl a and Chl b during storage at 10°C and 15°C, particularly after 28 and 42 days (Figure 2C). By contrast, there were no observable changes in the peel color, CCI values, and Chl content of fruit during storage at 5°C, 20°C, and 25°C throughout the entire storage duration.

### On-Tree Peel Degreening

**Figure 3:**
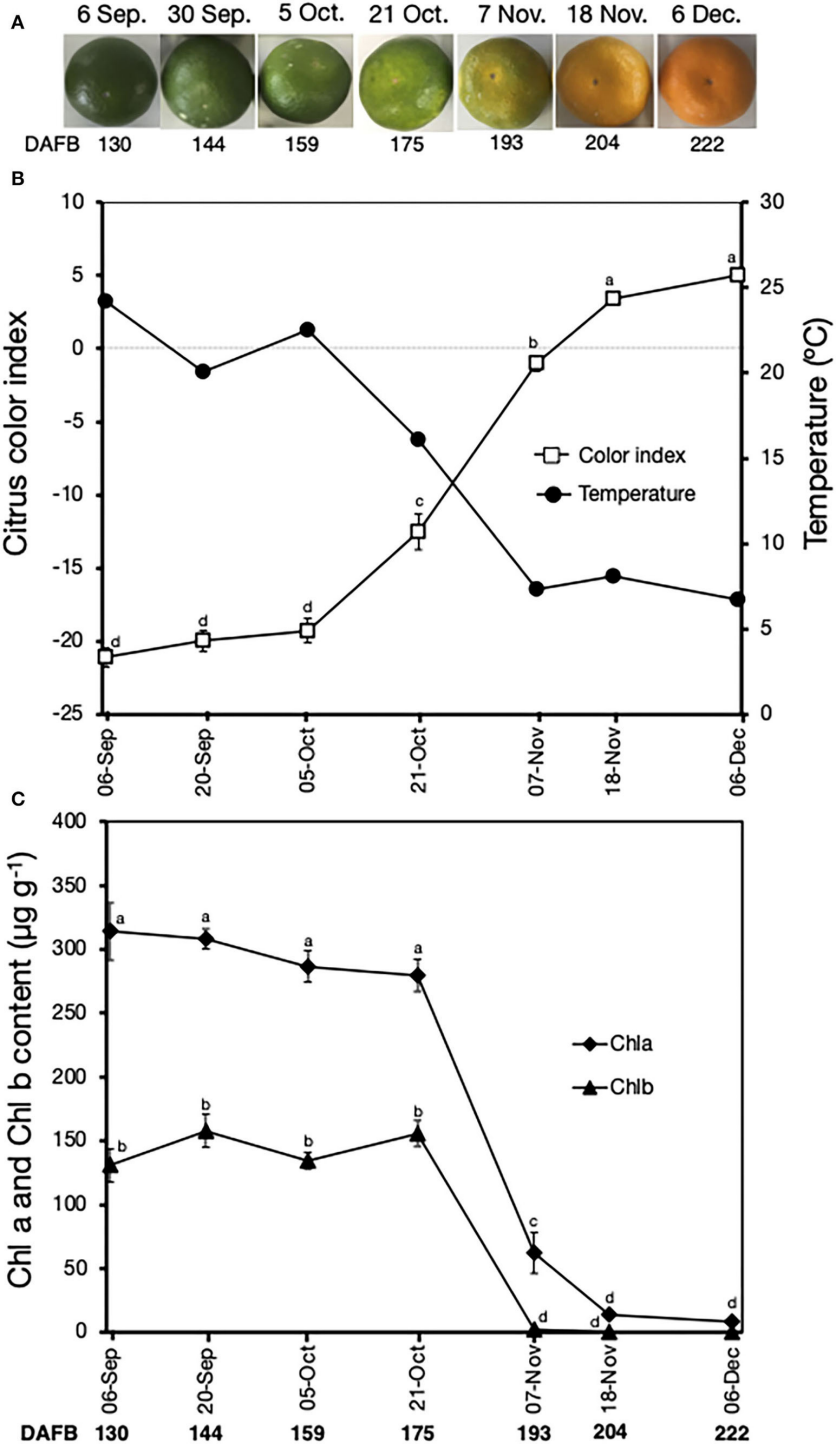
Peel color changes in Satsuma mandarin fruit during on-tree maturation. **(A)** Appearance of fruit during on-tree maturation. **(B)** Changes in citrus color index and minimum field temperatures. Data for temperature were accessed from the website of Japan Meteorological Agency (http://www.data.jma.go.jp/obd/stats/etrn/view/daily_s1.php?prec_no=72&block_no=47891&year=2014&month=12&day=&view=p1). **(C)** Changes in the peel content of chlorophyll a and chlorophyll b. Data points represent the mean (±SE) of five fruit and different letters indicate significant differences in ANOVA (Tukey's test, *P* < 0.05). Samples for on-tree assessments were collected in 2017. DAFB, days after full bloom.

Peel color changes were also monitored in fruit harvested at seven progressive stages during on-tree maturation. The peel color of the fruit gradually changed from the initial green in early-September to a uniform orange in early-December (Figure 3A). The CCI also increased (Figure 3B), initially at a slow rate between September 6 and October 5 (from −21 to −19), and then rapidly between October 5 and December 6 (from −19 to 5). These peel color changes were then aligned to changes in daily minimum orchard temperatures, which showed a gradual decline from 24.2°C on September 6 to 6.7°C on December 6. Fruit remained green with minimal changes in CCI values from September 6 to October 5, during which temperatures were ≥20°C (Figure 3B). Interestingly, peel degreening was initiated from October 21 onwards when the daily minimum temperatures were ≤ 16°C. Peel Chl a and Chl b content also showed similar trends (Figure 3C); they remained unchanged until October 21 and decreased rapidly afterward to minimal and undetectable levels on December 6 for Chl b and Chl a, respectively.

### Differential Gene Expression During Postharvest Peel Degreening

**Figure 4:**
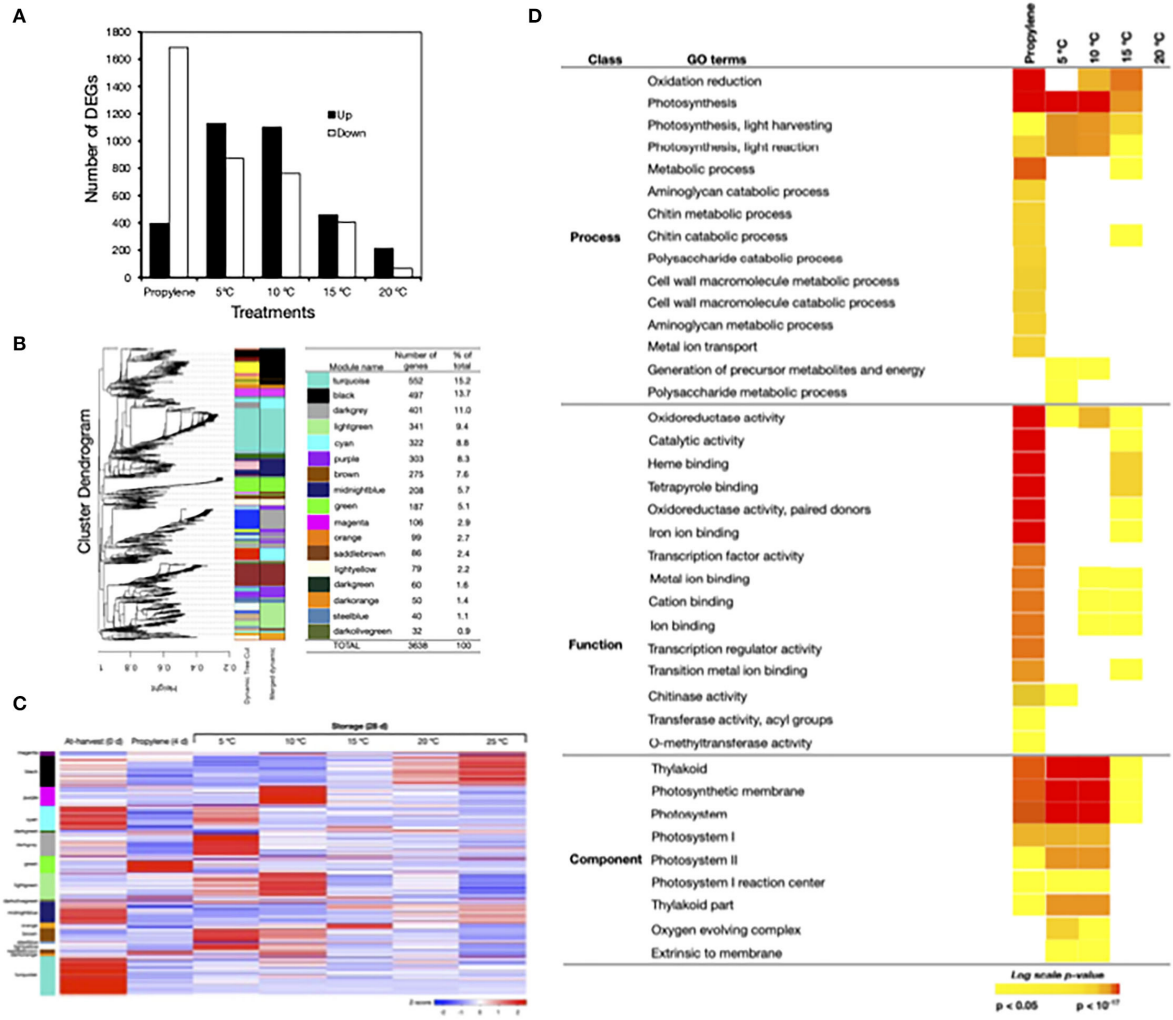
Transcriptomic changes in the flavedo of Satsuma mandarin fruit in response to propylene and different storage temperatures. **(A)** Number of genes that were differentially expressed in response to propylene treatment and storage at 5°C, 10°C, 15°C, and 20°C. Propylene effect was determined using at-harvest (0 days) samples as a control, whereas the control samples for storage temperature tests included fruit at 25°C. **(B)** Weighted gene coexpression network analysis (WGCNA) of differentially expressed genes (DEGs) identified from the flavedo of Satsuma mandarin fruit treated with propylene or stored at different temperatures. The left panel shows the cluster dendrogram with the major tree branches. The right panel shows the different colors assigned to each one of the 17 modules obtained after implementing the tree cut line (0.15) to merge close modules and the number of genes in each module. **(C)** Heatmap showing the expression measures of genes in each WCGNA module at harvest (0 days), after 4 days propylene treatment and 28 days storage at the specified temperatures. **(D)** Selected gene ontology (GO) terms that were enriched among the DEGs that responded to propylene treatment or storage at 5°C, 10°C, and 15°C. Color panels display the *P*-values of the respective GO enrichment terms.

As the above findings demonstrated that postharvest peel degreening in Satsuma mandarin fruit was induced by either propylene treatment or storage at moderately low temperatures, we examined the transcriptional changes involved *via* RNA-Seq analysis (Figure 4). This analysis identified 3,694 DEGs, 780 of which responded exclusively to propylene, 1,580 to low temperature (5, 10, 15, and 20°C), and 1,334 to both propylene and low temperature (Supplementary Tables 2–4). Overall, more genes were downregulated than upregulated by propylene treatment, while the reverse pattern was observed in response to either 5°C, 10°C, 15°C, or 20°C (Figure 4A). Subsequent hierarchical clustering of the total DEGs against temporal expression patterns in at-harvest, propylene-treated, and stored samples outlined 17 major modules (Figure 4B). Heatmapping of the DEGs indicated that the two major modules, “turquoise” (15.2%) and “black” (13.7%), comprised mostly downregulated genes (Figure 4C). The “dark gray” module genes (11%) were downregulated by propylene but upregulated during storage at 5°C. Genes in the “light green” module (9.4%) did not respond to propylene treatment, but they were upregulated at 5°C and 10°C, while “cyan” module genes (8.8%) were downregulated by propylene and during storage regardless of temperature. The “purple” (8.3%) and “orange” (2.7%) modules comprised genes that were exclusively upregulated at 10°C and 15°C, respectively. The remaining minor modules comprised genes that displayed varied expression patterns in response to propylene treatment and storage temperature.

To further examine the transcriptome changes induced during postharvest peel degreening, we performed a GO term enrichment analysis with DEGs nested into each treatment. Notable GO terms that were significantly enriched by propylene treatment and storage at 5°C, 10°C, and 15°C included “photosynthesis” and its components, namely “thylakoid,” “photosystem,” and “photosynthetic membrane” (Figure 4D). In addition, genes associated with “oxidoreductase” activity were significantly enriched by propylene treatment and storage at 5°C, 10°C, and 15°C. It is worth mentioning that no GO terms were significantly enriched at 20°C.

### Postharvest Expression Patterns of Chl Degradation-Related Genes

**Figure 5:**
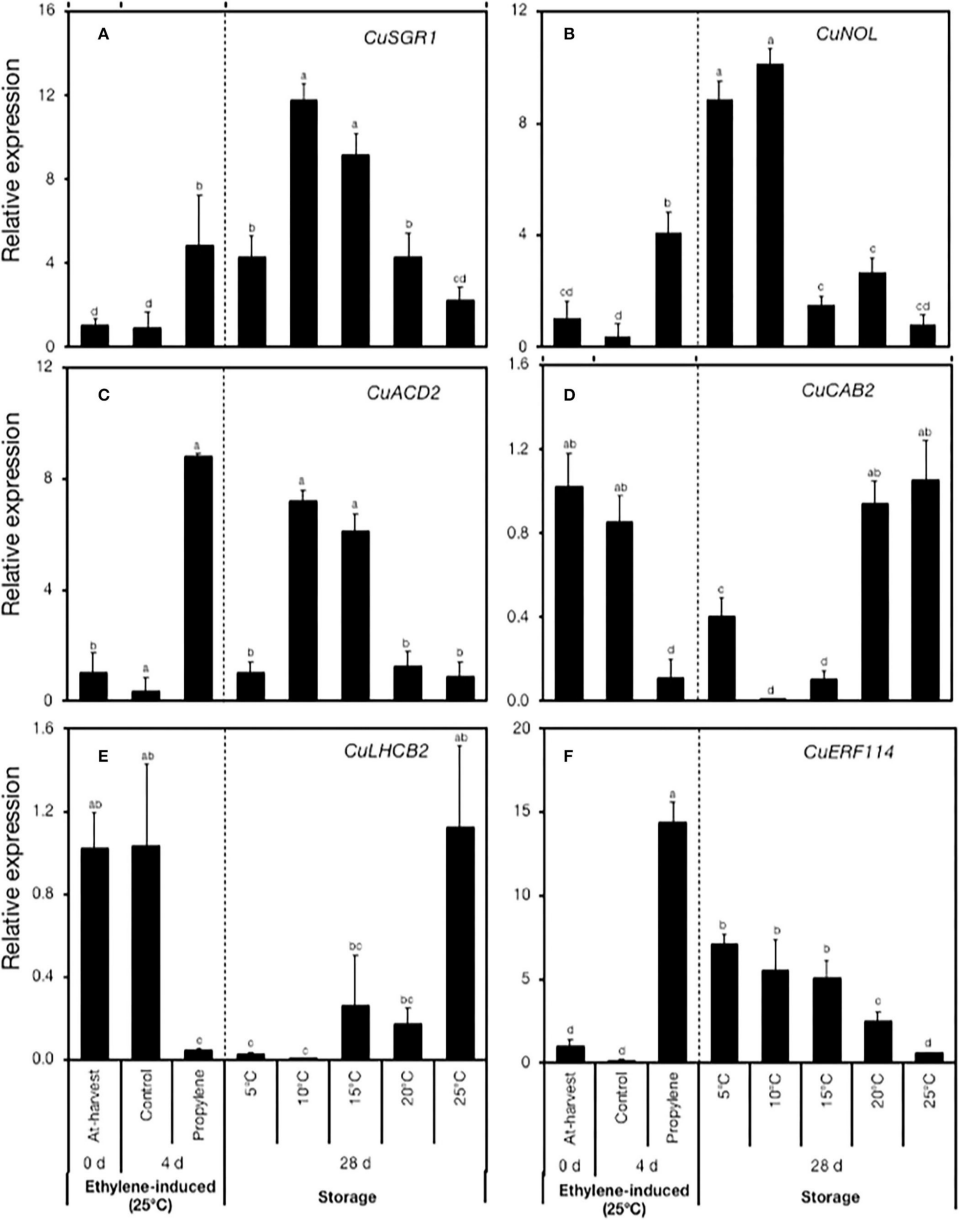
RT-qPCR analysis of the expression of genes associated with chlorophyll degradation and a transcription factor selected from the RNA-Seq data. **(A)** *CuSGR1* (*Ciclev10021651m*.g), **(B)** *CuNOL* (*Ciclev10008039m*.g), **(C)** *CuACD2* (*Ciclev10026248m*.g), **(D)** *CuCAB2* (*Ciclev10016286m*.g), **(E)** *CuLHCB2* (*Ciclev10016280m*.g), and **(F)** *CuERF114* (*Ciclev10032575m*.g). Data points represent the mean (±SE) of three fruit and different letters indicate significant differences in ANOVA (Tukey's test, *P* < 0.05).

To validate the RNA-Seq data, we used RT-qPCR to examine the expression of five genes that have been previously associated with Chl degradation in citrus fruit and plants at large. The expression of *CuSGR1, CuNOL*, and *CuACD2* increased in response to propylene treatment (Figures 5A–C), but varied patterns were displayed during storage. *CuSGR1* expression increase was significantly higher at 10°C and 15°C (9–12-fold) than at 5°C and 20°C (4-fold), while changes at 25°C were insignificant (Figure 5A). The expression of *CuNOL* increased by 9- and 10-fold at 5°C and 10°C, respectively (Figure 5B), but changes at 15°C, 20°C, and 25°C were insignificant. *CuACD2*, on the contrary, was upregulated at 10°C and 15°C (6–7-fold), while only minimal changes were registered at 5°C, 20°C, and 25°C (Figure 5C). Two gene-encoding photosystem proteins, namely, *CuCAB2* and *CuLHCB2*, were downregulated both in response to propylene treatment and during storage at low temperatures relative to 25°C (Figures 5D,E). In particular, *CuCAB2* was downregulated at 5°C, 10°C, and 15°C (Figure 5D), whereas *CuLHCB2* was downregulated at 5°C, 10°C, 15°C, and 20°C (Figure 5E). Finally, the expression of an ethylene response TF-encoding gene *CuERF114* increased both in response to propylene treatment and during low-temperature storage (5°C, 10°C, 15°C, and 20°C) but not at 25°C (Figure 5F).

### Gene Expression During on-Tree Peel Degreening

**Figure 6:**
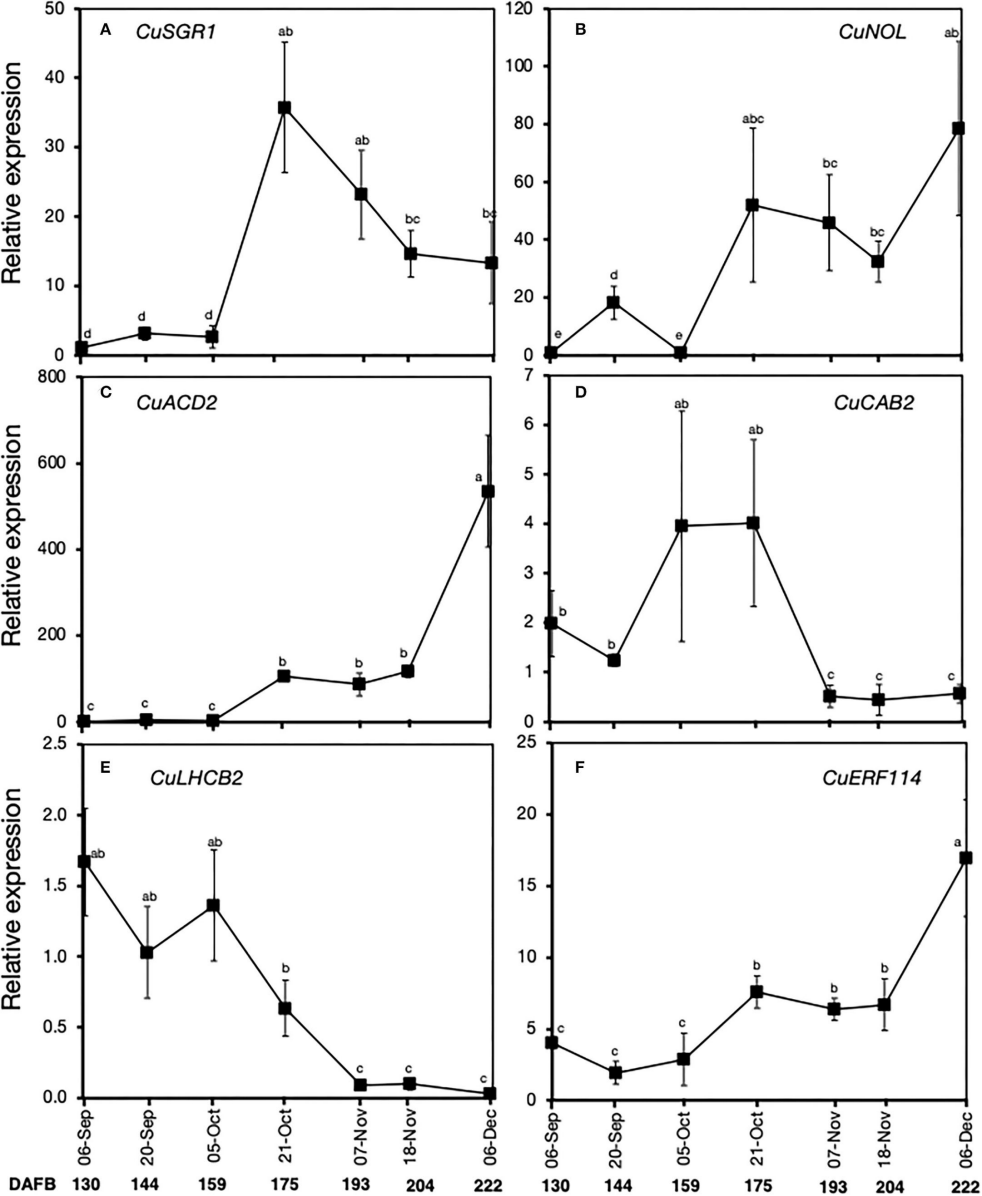
Relative expression levels of selected genes associated with chlorophyll degradation and a transcription factor in the flavedo of Satsuma mandarin fruit during on-tree peel degreening. Values below the horizontal axis indicate the average minimum environmental temperature recorded on the specified date. **(A)** *CuSGR1* (*Ciclev10021651m*.g), **(B)** *CuNOL* (*Ciclev10008039m*.g), **(C)** *CuACD2* (*Ciclev10026248m*.g), **(D)** *CuCAB2* (*Ciclev10016286m*.g), **(E)** *CuLHCB2* (*Ciclev10016280m*.g), and **(F)** *CuERF114* (*Ciclev10032575m*.g). Data points represent the mean (±SE) of three fruit and different letters indicate significant differences in ANOVA (Tukey's test, *P* < 0.05). Samples for on-tree assessments were collected in 2017. DAFB, days after full bloom.

Quantitative real-time PCR analysis was also used to further examine the expression patterns of the above six genes during on-tree peel degreening. There was an increase in the expression of *CuSGR1, CuNOL*, and *CuACD2*, particularly on October 21 and subsequent harvest dates (Figures 6A–C), which coincided with the onset of peel degreening. *CuERF114* also displayed a steady increase in expression from October 21 to December 6 (Figure 6F). On the contrary, there was a sharp and constant decrease in the expression of *CuCAB2* on November 7 (Figure 6D) and *CuLHCB2* on October 21 (Figure 6E).

### Ethylene-Specific and Low-Temperature-Specific Genes: Postharvest vs. on-Tree Expression Patterns

**Figure 7:**
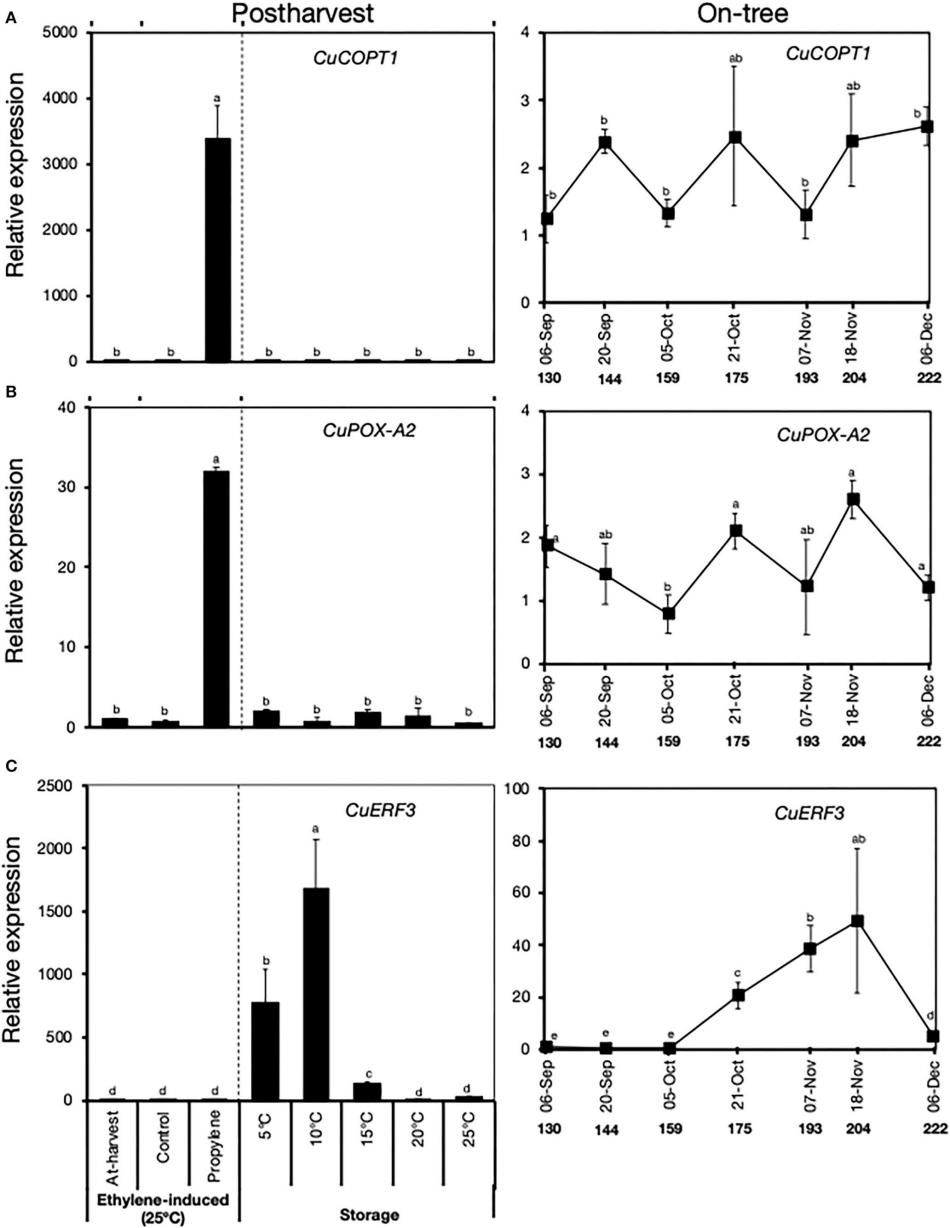
Relative expression levels of ethylene-specific **(A,B)** and low temperature-specific **(C)** genes during postharvest and on-tree peel degreening in Satsuma mandarin fruit. Values below the horizontal axis of on-tree graphs indicate the days after full bloom. *CuCOPT1* (*Ciclev10030036m.g*), *CuPOX-A2* (*Ciclev10015790m*.g), and *CuERF3* (*Ciclev10009593m*.g). Data points represent the mean (±SE) of three fruit and different letters indicate significant differences in ANOVA (Tukey's test, *P* < 0.05).

Results of our RNA-Seq analysis revealed the existence of two distinct groups of genes that were differentially expressed during peel degreening. The first group of genes was influenced by propylene treatment but not by low storage temperature, and hence they were considered ethylene-specific (Supplementary Table 2). The second group of genes (low temperature-specific) did not respond to propylene, but they were influenced by low storage temperatures (Supplementary Table 3). We used RT-qPCR analysis to compare the expression patterns of two ethylene-specific genes and one low temperature-specific gene during postharvest and on-tree peel degreening (Figure 7). During postharvest peel degreening, the two ethylene-specific genes, *copper transport protein 1* (*CuCOPT1*) involved in copper homeostasis and *peroxidase* (*CuPOX-A2*) involved in oxidative stress response, were upregulated by propylene treatment, whereas minimal changes in expression were recorded during storage (Figures 7A,B). Both *CuCOPT1* and *CuPOX-A2* displayed minimal changes in expression during on-tree peel degreening (Figures 7A,B). Conversely, *CuERF3* expression showed little changes in response to propylene treatment but increased highly during storage at 5°C, 10°C, and 15°C, but not at 20°C or 25°C (Figure 7C). The expression of *CuERF3* steadily increased during on-tree peel degreening from October 21, peaked on November 18, and then dropped sharply on December 6 (Figure 7C). Together, these findings demonstrated that the transcriptional changes associated with on-tree peel degreening were similar with those of low temperature-induced peel degreening during postharvest storage. Equally, on-tree peel degreening changes appeared to be different from ethylene-induced ones.

## Discussion

Peel degreening is a critical part of fruit ripening in many citrus fruits (Iglesias et al., 2007), and there is an ongoing effort, both for physiological and commercial reasons, to deduce the regulatory mechanisms involved. In the present study, treatment with propylene, a well-known ethylene analog (McMurchie et al., 1972), triggered rapid peel degreening and the associated loss of Chl pigments in Satsuma mandarin fruit (Figure 1). These findings are consistent with previous reports on Satsuma mandarins (Morales et al., 2020) and other citrus fruits (Rodrigo and Zacarias, 2007; Shemer et al., 2008; Mayuoni et al., 2011), where the application of exogenous ethylene was shown to accelerate ripening-related peel color changes. The mechanisms and pathways involved in citrus peel degreening in response to exogenous ethylene sources have been well-characterized to date (Shemer et al., 2008; Alós et al., 2014; Yin et al., 2016). However, the causal factors of natural peel degreening remain elusive, as mature citrus fruit are non-climacteric, producing only minute endogenous ethylene amounts (Katz et al., 2004). Indeed, ethylene production levels were undetectable both in propylene-treated and stored samples (data not shown), confirming the non-climacteric nature of the Satsuma mandarin fruit used in this study.

Postharvest storage at 10°C and 15°C clearly stimulated green color loss and a reduction in peel Chl content, whereas no changes were observed at 20°C and 25°C (Figure 2), which indicated that low temperatures are required for peel degreening to occur. Previous studies have also demonstrated that low/intermediate temperatures promote peel degreening in various citrus fruit species (Zhu et al., 2011; Tietel et al., 2012; Mitalo et al., 2020). The regulatory mechanisms for low temperature-triggered peel degreening are still unclear, but it has been suggested that ethylene signaling is involved; that is, either trace levels of System I ethylene produced by mature citrus fruit are physiologically active (Goldschmidt et al., 1993; Carmona et al., 2012b) or the ethylene sensitivity of mature citrus fruit likely increases during maturation (Alós et al., 2014). However, we recently demonstrated that repeated treatments of lemon fruit with 1-methylclopropene (1-MCP) to inhibit ethylene signaling failed to abolish the accelerated peel degreening triggered during low-temperature storage. These findings indicate that ethylene signaling is not functional during natural peel degreening, at least in mature lemon fruit. In this study, we attempted to replicate this finding in Satsuma mandarins, but 1-MCP treatments surprisingly stimulated massive ethylene production and subsequent peel browning (Supplementary Figure 1).

To delve into the mechanism of how low storage temperatures induced peel degreening in Satsuma mandarins, we analyzed the transcriptomic changes involved relative to those induced by ethylene. This analysis enabled us to identify a distinct set of genes that were exclusively regulated by low temperature, and ethylene signaling triggered by propylene treatment had no influence on their expression (Figure 4C, Supplementary Table 3). These low temperature-specific genes provided us with the first indication that the mechanisms involved in low-temperature promotion of peel degreening are independent of ethylene influence. Additionally, there was a second distinct set of genes that were exclusively regulated by the ethylene signal triggered by propylene treatment (Figure 4C, Supplementary Table 2). The finding that these ethylene-specific genes showed insignificant changes in expression during storage further suggests that ethylene signaling was not functional in stored Satsuma mandarins. Thus, even though the third group of genes, including those associated with Chl degradation, responded to both propylene treatment and low storage temperatures (Figure 5, Supplementary Table 4), it appears that distinct and independent regulatory mechanisms are involved. Indeed, Mitalo et al. (2020) demonstrated that *ClLHCB2* was downregulated by both ethylene treatment and low storage temperatures during degreeening in mature lemons; however, its downregulation by low temperature was not inhibited by repeated 1-MCP treatments, confirming that the observed expression changes were independent of ethylene.

On-tree peel degreening and the associated reduction in Chl content typically coincided with gradual autumnal drops in environmental temperature (Figure 3). The peel color changes and associated gene expression were particularly initiated when ambient temperatures in the orchard location had decreased to below 16°C (Figures 3, 6), which also agrees with previous findings in other citrus genotypes (Manera et al., 2012, 2013; Conesa et al., 2019), indicating that low ambient temperatures are required for normal degreening during on-tree maturation. Interestingly, the two ethylene-specific genes presented in this study (*CuCOPT1* and *CuPOX-A2*) showed insignificant expression changes during on-tree peel degreening (Figures 7A,B), further alluding to a non-functional ethylene signaling pathway in mature Satsuma mandarin fruits. In lemons, however, *ClCOPT1* expression levels were undetectable by RNA-Seq while *ClPOX-A2* was upregulated highly by ethylene treatment and slightly by low storage temperatures (Mitalo et al., 2020), and this highlights fruit species-related differences in gene expression patterns during peel degreening. Nevertheless, the low temperature-specific expression pattern displayed by *CuERF3* (Figure 7C) was also reported in lemon fruit during on-tree peel degreening as environmental temperatures decreased. These findings clearly suggest that low temperature plays a role in modulating on-tree peel degreening in Satsuma mandarins likely in an ethylene-independent manner.

The transcriptomic data further indicated that many genes were differentially expressed during storage at 5°C (Figure 4A), yet no peel degreening changes took place (Figure 2). A similar observation was also reported in our previous study of lemon fruit (Mitalo et al., 2020). While the cause of this anomaly is unclear, it could possibly be due to the slow protein synthesis rate and/or low activity of peel degreening-associated enzymes at 5°C (Yun et al., 2012).

Previous studies have demonstrated the correlation between Chl degradation and the expression of *CLH* genes during ethylene-induced degreening in various citrus genotypes (Jacob-Wilk et al., 1999; Yin et al., 2016). However, transcript levels of *CLH* do not increase during peel degreening in non-treated fruit both on and off the tree (Jacob-Wilk et al., 1999; Mitalo et al., 2020), raising uncertainties about its role. In this study, all the identified *CLH* genes, including *CuCLH1*, were downregulated both in response to ethylene and low storage temperatures and displayed minimal expression changes during on-tree peel degreening (Supplementary Figure 2, Supplementary Table 2, Supplementary Table 3). Therefore, it is possible that, in Satsuma mandarin fruit, CLH enzyme activity is either not involved in Chl degradation or regulated at a different level other than transcription.

Besides Chl degradation, the other important process that contributes to peel degreening is carotenoid metabolism (Kato, 2012). While the carotenoid content and profiles were not quantified in this study, RNA-Seq analysis identified many biosynthetic genes that were differentially expressed, and subsequent RT-qPCR analysis confirmed that most of them, including *CuPSY1, CuLCYb2a*, and *CuNCED5*, were upregulated by both propylene treatment and low storage temperatures (Supplementary Figure 3). Increased expression of carotenoid biosynthetic genes in response to either ethylene signaling, or low temperature storage has also been reported during peel degreening in various citrus fruits (Rodrigo and Zacarias, 2007; Matsumoto et al., 2009; Mitalo et al., 2020).

## Conclusion

This study investigated the roles of ethylene and low temperature in fruit ripening-related peel degreening in Satsuma mandarin fruit during on-tree maturation and postharvest storage. Transcriptomic data unveiled distinct sets of genes that were regulated by ethylene signaling and/or low temperature. A comparison of the expression patterns of these genes provides a clear indication that low temperature promotes peel degreening in Satsuma mandarin fruit, most likely *via* an ethylene-independent mechanism.

## Data Availability Statement

The datasets presented in this study can be found in online repositories. The names of the repository/repositories and accession number(s) can be found below: DNA Data Bank of Japan (DDBJ) database (DRR376678–DRR376696).

## Author Contributions

OM, WA, and YK conceived and designed the study. OM and WA did most of the experiments with close supervision from YK, TA, and KU. KU performed RNA-Seq analysis. SK and HE provided technical assistance. OM wrote the first draft of the manuscript which was substantially improved by SK and HE. All authors have read and approved the submitted version. All authors contributed to the article and approved the submitted version.

## Funding

OM is a JSPS International research Fellow (Graduate School of Life and Environmental Sciences, University of Tsukuba). This study was supported in part by a Grant-in-Aid for Scientific Research (grant no. 20H02977) by the Japan Society for the Promotion of Science, Japan (JSPS).

## Conflict of Interest

WA is employed by Del Monte Kenya Ltd. The remaining authors declare that the study was conducted in the absence of any commercial or financial relationships that could be construed as a potential conflict of interest.

## Publisher's Note

All claims expressed in this article are solely those of the authors and do not necessarily represent those of their affiliated organizations, or those of the publisher, the editors and the reviewers. Any product that may be evaluated in this article, or claim that may be made by its manufacturer, is not guaranteed or endorsed by the publisher.

## Footnotes

^1^ https://www.maff.go.jp/j/tokei/kouhyou/sakumotu/sakkyou_kazyu/index.html#y

^2^ http://bioinfo.ut.ee/primer3-0.4.0/

^3^ www.r-project.org
