# Supplementary material for: Examining the Role of Low Temperature in Satsuma Mandarin Fruit Peel Degreening via Comparative Physiological and Transcriptomic Analysis
Source: Front Plant Sci. 2022 Jul 13;13:918226. doi: 10.3389/fpls.2022.918226 (PMC9328020; doi:10.3389/fpls.2022.918226)
Supplement: Supplementary file 1 [file Data_Sheet_1.ZIP › Supplementay Material_1/Supplementary Figure 3.pptx]

## Slide 1
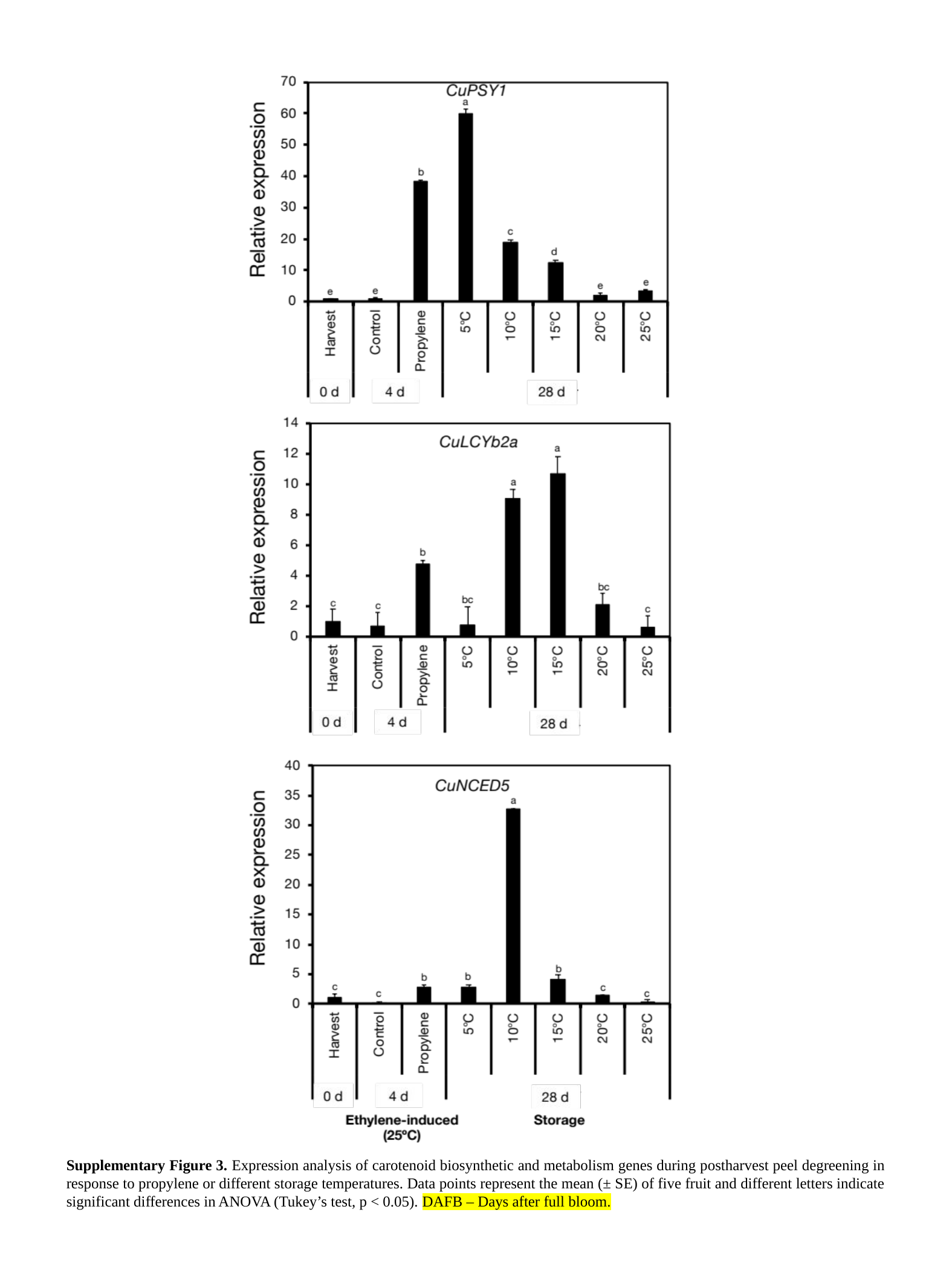

Supplementary Figure 3. Expression analysis of carotenoid biosynthetic and metabolism genes during postharvest peel degreening in response to propylene or different storage temperatures. Data points represent the mean (± SE) of five fruit and different letters indicate significant differences in ANOVA (Tukey’s test, p < 0.05). DAFB – Days after full bloom.
