# Supplementary material for: Examining the Role of Low Temperature in Satsuma Mandarin Fruit Peel Degreening via Comparative Physiological and Transcriptomic Analysis
Source: Front Plant Sci. 2022 Jul 13;13:918226. doi: 10.3389/fpls.2022.918226 (PMC9328020; doi:10.3389/fpls.2022.918226)
Supplement: Supplementary file 1 [file Data_Sheet_1.ZIP › Supplementay Material_1/Supplementary Figure 2.pptx]

## Slide 1
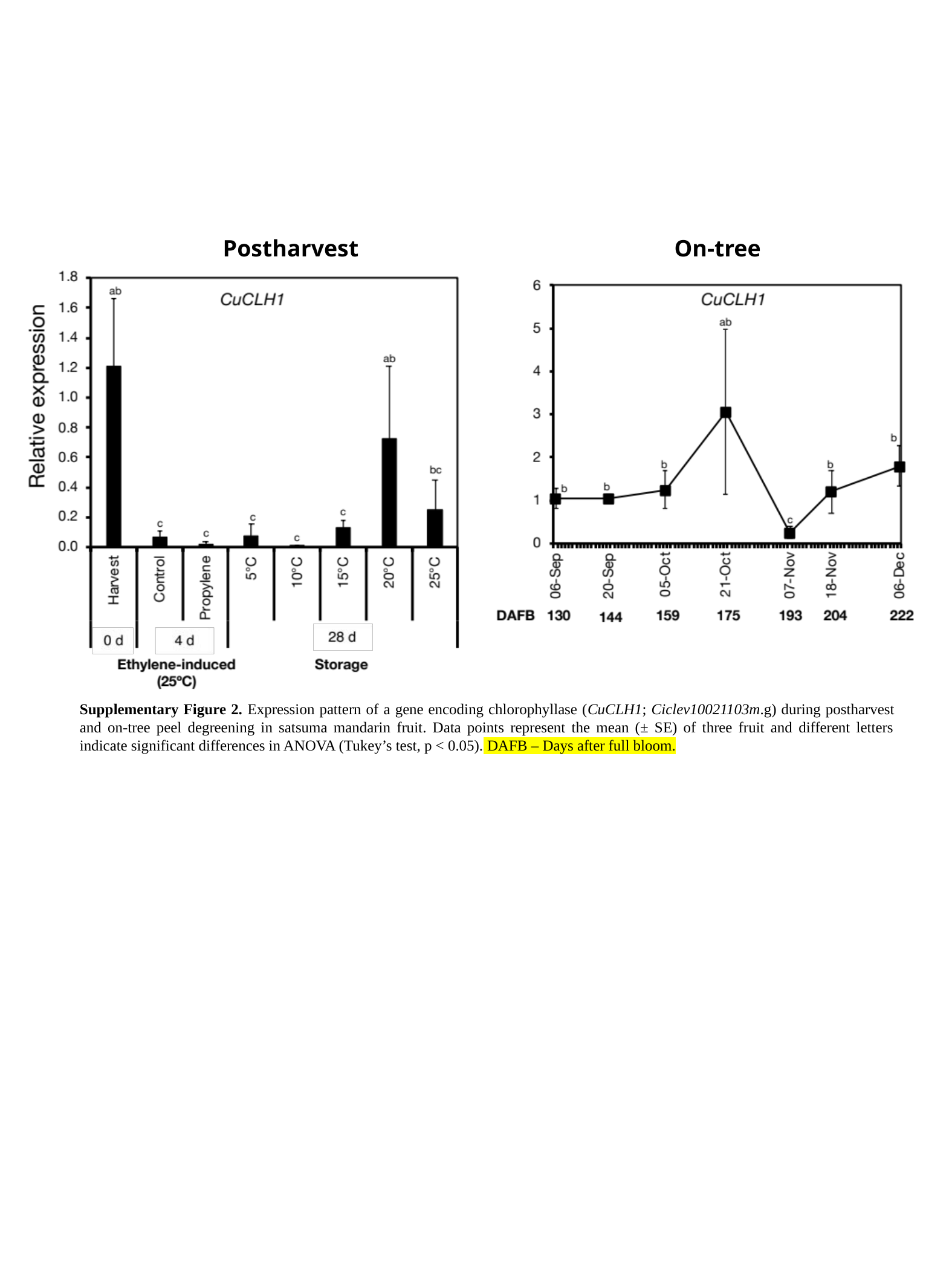

Postharvest
On-tree
Supplementary Figure 2. Expression pattern of a gene encoding chlorophyllase (CuCLH1; Ciclev10021103m.g) during postharvest and on-tree peel degreening in satsuma mandarin fruit. Data points represent the mean (± SE) of three fruit and different letters indicate significant differences in ANOVA (Tukey’s test, p < 0.05). DAFB – Days after full bloom.
