# Supplementary material for: Examining the Role of Low Temperature in Satsuma Mandarin Fruit Peel Degreening via Comparative Physiological and Transcriptomic Analysis
Source: Front Plant Sci. 2022 Jul 13;13:918226. doi: 10.3389/fpls.2022.918226 (PMC9328020; doi:10.3389/fpls.2022.918226)
Supplement: Supplementary file 1 [file Data_Sheet_1.ZIP › Supplementay Material_1/Supplementary Table 1_mikan.docx]

**Supplementary Table 1. Oligonucleotide sequences of primers used for RT-qPCR analysis in this study.**

| **Name** | **Gene ID** | **Description** |  | **Sequence (5' to 3')** |
| --- | --- | --- | --- | --- |
| *CuSGR1* | Ciclev10021651m.g | Stay green 1 | 5' to 3’ | GAGTTGAAGCAACCACAACC |
|  |  |  | 3’ to 5’ | AGTCTTGGGGACAACACACA |
| *CuNOL* | Ciclev10008039m.g | NYC1-like | 5' to 3’ | TGCACTCGTGAGGCTATGC |
|  |  |  | 3’ to 5’ | GCTGTATGCACCCCAACTTTA |
| *CuACD2* | Ciclev10026248m.g | Accelerated cell death 2 | 5' to 3’ | CAAGTGACGCTTGCTGGATA |
|  |  |  | 3’ to 5’ | CTTCCCGTTGGTACCTTCAA |
| *CuCAB2* | Ciclev10016286m.g | Chlorophyll A-B binding protein 2 | 5' to 3’ | GTTGGGAGCTCTTGGATGC |
|  |  |  | 3’ to 5’ | CATCAATACCACCTGGCAA |
| *CuLHCB2* | Ciclev10016280m.g | Light harvesting complex 2 | 5' to 3’ | CTTCTCTGAAGGTGGCCTTG |
|  |  |  | 3’ to 5’ | CAAGTGGGTCAAAAGCACCAC |
| *CuERF114* | Ciclev10032575m.g | Ethylene response factor 114 | 5' to 3’ | CCGCTACAACTTCACAGCAA |
|  |  |  | 3’ to 5’ | GAGACGACGTCGCAGAAGA |
| *CuCOPT1* | Ciclev10030036m.g | Copper transporter 1 | 5' to 3’ | TTCTGGGGCACAAATACTGA |
|  |  |  | 3’ to 5’ | CCAATCCTGAAGGCGTACAT |
| *CuPOX-A2* | Ciclev10015790m.g | Peroxidase | 5' to 3’ | GTGATGCATCGATTTTGCTG |
|  |  |  | 3’ to 5’ | ATTACCAAAGCAACGGATCG |
| *CuERF3* | Ciclev10009593m.g | Ethylene response factor 3 | 5' to 3’ | GAAACAGCTGAAGACGCTGC |
|  |  |  | 3’ to 5’ | AGGCCATGTAGCACCTATGC |
| *CuCLH1* | Ciclev10021103m.g | Chlorophyllase | 5' to 3’ | ATCATCTCCATCCTCACCAC |
|  |  |  | 3’ to 5’ | CTGAGGAGCAACAACGATGA |
| *CuPSY1* | Ciclev10011841m.g | Phytoene synthase | 5' to 3’ | GCACCCGGCTAGCATATCT |
|  |  |  | 3’ to 5’ | GAGGTGCAACTTAGGGGTGA |
| *CuLCYb2a* | Ciclev10028245m.g | Lycopene cyclase 2a | 5' to 3’ | GTGATCATCATTGGCACTGGA |
|  |  |  | 3’ to 5’ | AAGTCATCGGCCAAGTTTTG |
| *CuNCED5* | Ciclev10014639m.g | 9-cis-epoxycarotenoid dioxygenase 5 | 5' to 3’ | ACCCACGTGTCCAAATTAGC |
|  |  |  | 3’ to 5’ | ACTTGCGCTTCCGTTTCC |
