# Supplementary material for: Examining the Role of Low Temperature in Satsuma Mandarin Fruit Peel Degreening via Comparative Physiological and Transcriptomic Analysis
Source: Front Plant Sci. 2022 Jul 13;13:918226. doi: 10.3389/fpls.2022.918226 (PMC9328020; doi:10.3389/fpls.2022.918226)
Supplement: Supplementary file 1 [file Data_Sheet_1.ZIP › Supplementay Material_1/Supplementary Figure 1.pptx]

## Slide 1
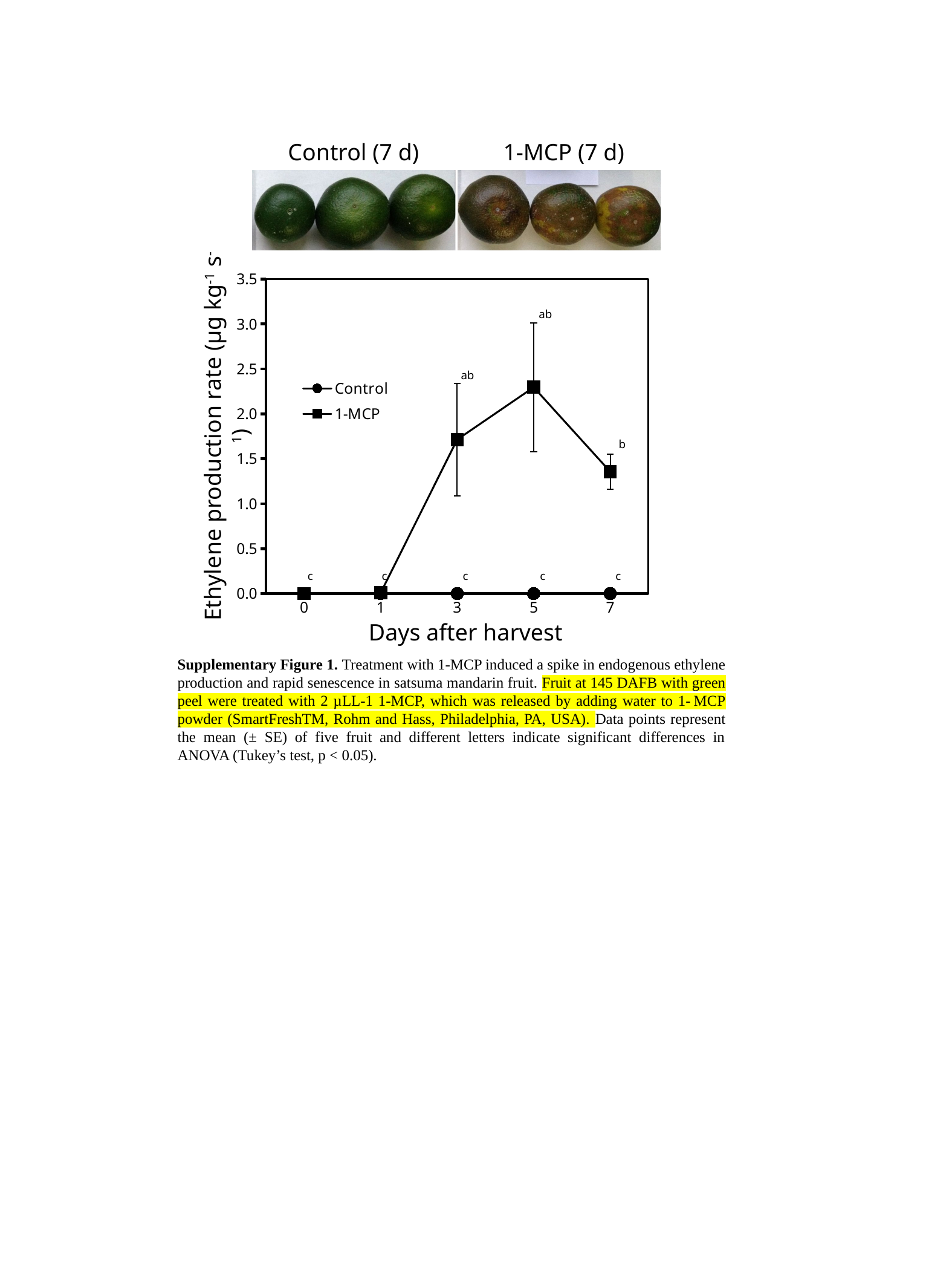

Control (7 d)
1-MCP (7 d)
### Chart
| Category | Control | 1-MCP |
|---|---|---|
| 0 | 0.0 | 0.0 |
| 1 | 0.0 | 0.007362658883677674 |
| 3 | 0.0 | 1.713996484579948 |
| 5 | 0.0 | 2.295776188120552 |
| 7 | 0.0 | 1.3570230719964236 |ab
ab
Ethylene production rate (µg kg-1 s-1)
b
c
c
c
c
c
Days after harvest
Supplementary Figure 1. Treatment with 1-MCP induced a spike in endogenous ethylene production and rapid senescence in satsuma mandarin fruit. Fruit at 145 DAFB with green peel were treated with 2 µLL-1 1-MCP, which was released by adding water to 1- MCP powder (SmartFreshTM, Rohm and Hass, Philadelphia, PA, USA). Data points represent the mean (± SE) of five fruit and different letters indicate significant differences in ANOVA (Tukey’s test, p < 0.05).
